# Why Sexually Transmitted Infections Tend to Cause Infertility: An Evolutionary Hypothesis

**DOI:** 10.1371/journal.ppat.1004111

**Published:** 2014-08-07

**Authors:** Péter Apari, João Dinis de Sousa, Viktor Müller

**Affiliations:** 1 MTA-ELTE Theoretical Biology and Evolutionary Ecology Research Group, Eötvös Loránd University and the Hungarian Academy of Sciences, Budapest, Hungary; 2 Laboratory for Clinical and Epidemiological Virology, Department of Microbiology and Immunology, Rega Institute for Medical Research, Katholieke Universiteit Leuven, Leuven, Belgium; 3 Centro de Malária e Outras Doenças Tropicais and Unidade de Microbiologia, Instituto de Higiene e Medicina Tropical, Universidade Nova de Lisboa, Lisboa, Portugal; 4 Parmenides Center for the Conceptual Foundations of Science, Pullach/Munich, Germany; The Fox Chase Cancer Center, United States of America

In this Opinion piece we argue that the tendency of sexually transmitted infections (STIs) to cause infertility is likely to reflect an evolutionary adaptation of the pathogens. We use an evolutionary perspective to understand how STI pathogens may benefit from reducing fertility in the host and what clues the mechanisms of pathogenesis can offer to the evolution of this ability. While we concentrate on human infections, we will also briefly discuss the broader context of STI-induced infertility in other species.

STIs are a common cause of human infertility worldwide (Box 1). While several nonsexually transmitted infections can also cause infertility (e.g., schistosomiasis, tuberculosis, leprosy [1]), these infections are typically associated with high overall virulence. In contrast, STIs tend to cause little mortality and morbidity; thus, the effect on fertility seems to be more “targeted” and specific. In addition, several STI pathogens are also associated with an increased risk of miscarriage and infant mortality (Box 1). Reduced fertility and an increased risk of complications during and following pregnancy both contribute to reduced reproductive success in the host—and may benefit the sexually transmitted pathogen by destabilizing partnerships and increasing promiscuity. The birth of a child has a strong positive effect on marital stability [2]; conversely, infertility often results in the break-up of a couple and a change of partners [3], and a childless couple may also have increased rates of extramarital sexual contacts [3]. An STI pathogen that causes infertility or miscarriage will therefore benefit from increased rates of partner exchange and promiscuity, which facilitates its transmission within the population [4]–[7]. Pregnancy also has a direct negative effect on sexual activity, which tends to decrease considerably in the months preceding and following childbirth [8]; the induction of infertility, miscarriage, and infant mortality can therefore facilitate STI transmission also, by avoiding or reducing this immediate effect. Finally, STIs are particularly likely to affect the “hubs” or “core” of the sexual network: the individuals of highest promiscuity, who may have a decisive role in the transmission dynamics [9]. Not only are highly promiscuous individuals exposed to a higher risk of acquiring STIs, but STIs may also actively generate hubs of transmission in a vicious circle of promiscuity and infertility: in traditional societies, “If a woman gets divorced because of infertility, she may have to turn to prostitution to survive” [10].

**Box 1. Major Human STIs That Affect Fertility/Reproduction**

The bacteria *Chlamydia trachomatis* and *Neisseria gonorrhoeae* are the major causes of pelvic inflammatory disease (PID), which in untreated women results in tubal factor infertility in 10%–40% of the cases [31]–[33] and increases the probability of ectopic pregnancy by more than 6-fold [31]. These two pathogens are responsible for more than 200 million new infections per year across the world [34]; with an estimated probability of PID up to 10%–25% in both infections [35], [36], the global burden of new cases of infertility due to these two pathogens may exceed 2 million per year. *N. gonorrhoeae* and, to a lesser extent, *C. trachomatis* have also been associated with reduced male fertility (reviewed in [17]), and both bacteria have been linked to increased risk of perinatal complications [37]. *Treponema pallidum*, the bacterial agent of syphilis, causes about 10 million new infections per year [34], and has a dramatic impact on pregnancy, with about one-third of untreated cases resulting in perinatal death (stillbirth or neonatal mortality) and another third in congenital infection (reviewed in [37]). *T. pallidum* alone is still responsible for a global burden of more than 300,000 perinatal deaths per year [38].

In addition to these major bacterial STDs, a number of other bacteria that can potentially be transmitted by sexual contact (e.g., *Gardnerella vaginalis*, *Mycoplasma hominis* and *Mycoplasma genitalium*, *Ureaplasma urealyticum*) have been associated with bacterial vaginosis, a frequent condition of disturbed vaginal microflora, which may increase the risk of PID [39], [40] and infertility [41] in women.

The sexually transmitted unicellular eukaryotic parasite *Trichomonas vaginalis* infects more than 270 million people per year [34] and increases the probability of pre-term birth, PID, female tubal, and male infertility [37], [42], [43]. Finally, some sexually transmitted viruses have also been implicated in reproductive health: genital herpes (caused by human herpesvirus types 1 and 2) may cause miscarriage and/or pregnancy complications [44] and is associated with reduced fertility in both sexes [45], [46]; human papillomaviruses may cause reduced sperm motility [47] and an increased risk of abortion [48]; human immunodeficiency virus infection adversely affects sperm quality [49].

While it seems clear that STI pathogens benefit from inducing infertility in the infected individuals, this is no direct proof for the adaptive evolution of this trait. To ensure sexual transmission, STIs tend to infect the reproductive tract, and infertility could potentially arise as a simple by-product of infection-related damage in the affected tissues [5], [11]. However, a growing body of evidence indicates the existence of targeted STI-induced mechanisms that affect fertility, but do not seem to improve the within-host replication of the pathogen or increase the contagiousness of the host (Box 2). Such mechanisms offer no direct benefit for the pathogen, and are therefore likely to have evolved for the indirect benefit afforded by manipulated host behaviour.

**Box 2. Specific Pathomechanisms of Infertility**

Here we list known mechanisms of STIs that reduce fertility of the infected individuals, but provide no apparent benefit for the within-host multiplication or per-contact transmissibility of the pathogens. In the absence of additional benefit, the existence of multiple mechanisms (in several cases, within the same species) indicates that the ability to cause infertility may itself have been selected for during the evolution of these infectious agents.

*N. gonorrhoeae* infects the nonciliated cells of the tubal mucosa, but destroys predominantly the uninfected ciliated cells that have a crucial role in the transport of fertilized eggs toward the uterus [50]. This effect is mediated by gonococcal lipopolysaccharides (LPS) that seem to have a targeted effect on the ciliated cells [51], and by up-regulating the production of tumour necrosis factor alpha [52]; the latter mechanism is selectively targeted to the uninfected ciliated cells by a protective anti-apoptotic effect exerted on infected cells by the pathogen [53]. *G. vaginalis* also seems to have a targeted mechanism to disable ciliated cells: filtrates of *G. vaginalis* cultures can arrest ciliary motility, indicating the presence of a soluble inhibitory factor [54]. Reduced ciliary activity is likely to decrease the probability of successful conception in both infections. *C. trachomatis* LPS, in turn, has a toxic effect on spermatozoa, which is orders of magnitude stronger than the effect of LPS from the non-STI bacteria that have been tested [55].

Chlamydia heat shock protein 60 (hsp60) induces apoptosis in trophoblasts [56]—cells of the placenta that are vital for normal fetal development [57]: this damage is likely to contribute to the adverse effect of *C. trachomatis* on pregnancy outcome [56]. The apoptotic effect is probably facilitated by the membrane-associated location of hsp60 in *C. trachomatis* (in contrast to the cytoplasmic location typical in other bacteria) [56], which may indicate a targeted adaptation of the pathogen. *N. gonorrhoeae* expresses a surface protein that mimics human chorionic gonadotropin, which has a vital role in pregnancy, and competitive binding to its receptor may contribute to increased risk of abortion [58].

The induction of crossreactive antibodies has also been implicated in the aetiology of infertility. Humoral immunity against chlamydial hsp60 is a predictor of autoreactive antibodies against human HSP60 and is associated with fallopian tube damage and increased risk of abortion (reviewed in [59]). In contrast, immunity against the hsp60 of *Escherichia coli* is not associated with an antibody response against the human HSP60 [60]; furthermore, hsp60 is selectively expressed at high levels also by the persistent form of *C. trachomatis*, which down-regulates the expression of most proteins [61]—these two observations are also consistent with a targeted pathomechanism. In addition, immunity against chlamydial hsp10 has also been linked to reduced fertility, and may have a distinct action mechanism by crossreacting with human HSP10, which has an important role as “early pregnancy factor” during pregnancy [62]. Finally, *C. trachomatis* infection increases the incidence of sperm-immobilizing antibodies in women [63], which may also contribute to infertility. Anti-sperm antibodies are also found in men with a history of infection, but their effect on fertility is unclear [64].

A further argument against the “accidental” nature of STI-induced infertility is based on the potential resource allocation trade-off between reproductive and immune function: infertility may redirect the energetic costs of reproduction to maintenance functions including immunity [12], and a parasite that induces infertility may therefore be exposed to a more potent immune response. If STI-associated infertility were a simple by-product of no adaptive value to the pathogen, then the selection pressure arising from enhanced immunity in sterilized hosts would drive the evolution of the pathogen towards the loss of the ability to induce infertility.

We therefore conclude that while STIs may have indeed been predisposed to cause infertility, the widespread existence of targeted pathomechanisms strongly suggests that this trait has been shaped by the adaptive evolution of the pathogens—which may have implications for treatments that would specifically target these mechanisms. If infertility serves the pathogen only by facilitating transmission, a treatment or vaccine that specifically targets a mechanism of infertility is not going to be opposed by the evolution of the pathogen within the host—which is typically the strongest level of selection. Therefore, the evolution of resistance or immune escape is much less likely against such targeted interventions than against currently used treatments that nearly always act by inhibiting the life cycle of the pathogen within the host [13] (e.g., emerging drug resistance is a serious concern in the management of gonorrhoea [14]). Based on these evolutionary considerations, we propose the development of novel drugs or vaccines that specifically target the molecular mechanisms of infertility.

Evolutionary thinking may provide further clues. While STIs are a major cause of infertility in women, their contribution to male infertility is relatively smaller [15]–[17], and this difference may also be understood in the context of pathogen evolution. Infertility destabilizes a couple, but this effect is not entirely symmetrical. If the male partner is sterile, the female may conceive a child from a single extramarital relationship, and the couple may be re-stabilized by the birth. However, if the female is sterile, the couple will remain childless, and the destabilizing effect persists. A further asymmetry is imposed by the direct effects of pregnancy, which only affect women. Finally, due to different parental investment of the two sexes, males have evolved a preference for higher baseline (uninfected) promiscuity in humans [18], as well as in most other species [19], which may allow for greater gain by manipulating female reproductive behaviour (in many males realized promiscuity will already be below their preference). These asymmetries imply that the benefits of infertility may be greater for the pathogen when imposed on a female, compared with a male host. Remarkably, the higher risk of infertility contrasts with a lower probability of symptomatic infection in women compared with men [20]: this further disconnection between general virulence (symptoms) and infertility may also hint at targeted pathomechanisms for the latter. However, we cannot completely exclude the simple nonselectionist explanation that ascending infection (associated with infertility) is more likely in the female than in the male reproductive system due to anatomical differences. Furthermore, STI pathogens present in semen tend to reduce sperm number and motility [21], [22], which, if sperm acts as a carrier, may relate reduced fertility to reduced infectivity and thereby inhibit evolution towards the former.

Finally, STI-induced infertility is not limited to human infections, but is a widespread phenomenon in animal STIs [4], [11]. Note that the effect of infertility on pathogen transmission is not limited to monogamous host species, but affects all species in which promiscuity, or a willingness to mate, decreases with successful conception. In a broader context, parasitic castration is a widespread phenomenon in which a parasite (not necessarily sexually transmitted) reduces host fertility to increase its own fitness, e.g., by exploiting the resources diverted from reproduction [23]. Animal STIs also provide further examples where the mechanism of infertility seems unrelated to the direct growth or transmissibility of the parasite [11].

While the idea that STIs may have evolved to induce infertility is not new [4]–[6], we have provided here a novel synthesis of the strong tendency of STIs to reduce fertility in their hosts, the evidence that infertility promotes STI transmission, and the widespread existence of targeted pathomechanisms that, taken together, suggest adaptive evolution of these traits. The public health implications of infertility and birth complications associated with STIs are huge. If not controlled, STI-induced infertility can reach staggering proportions: extremely high levels of STIs in some regions of sub-Saharan Africa in the first half of the 20th century were associated with rates of infertility exceeding 40% [24], and similar historical rates of STIs in the cities of Europe and North America may have implied a similar impact up to the beginning of the 20th century [25], [26]. Even today, STIs are an important cause in the estimated 9% global prevalence of infertility [27], and a cause of a terrible emotional burden [28] and loss of (unborn) lives. Evolutionary perspectives [6], [7], [29], [30] may help us understand these infections and may offer clues in the fight against them. We hope that our brief exposition of the “adaptive sterilization hypothesis” will prime further discussion and motivate new research to better define the limits of the validity of the hypothesis (Box 3).

**Box 3. Where Next?**

The arguments presented in this paper rely on indirect clues and inferences. While we believe that, taken together, they put forward a convincing case for the “adaptive sterilization hypothesis,” each line of evidence needs to be subjected to further tests and systematic analyses for validation. Below we propose some possibilities to investigate outstanding questions.

1. The association between STIs and infertility should be investigated systematically. The only study of this kind that we are aware of compiled a massive set of semiquantitative data on animal STIs [4] and found a somewhat higher probability of infertility in STIs compared with non-STIs in animals. However, the study also highlighted the problems of scarce and taxonomically biased data and of how to select a control set of nonsexually transmitted infections. As more data emerge, tests should contrast pairs of closely related pathogens, in which one of the pair is transmitted sexually while the other is transmitted via another route. However, even careful matching cannot control for the inherent bias that the majority of STIs infect the genital tract and are therefore predisposed to inflict damage there.
2. A more promising approach involves investigating the patterns of infertility among STIs that infect various host species. Based on the reproductive behaviour of the hosts (e.g., monogamy versus polygamy, continuous versus seasonal mating, parental care) and other life history traits of both the host and the pathogen (e.g., lifespan, duration of infection), the effect of infertility on promiscuity is likely to vary considerably, and may be predictable to some extent. A correlation between the predicted strength of this effect and the probability of infertility in different STIs would support the adaptive hypothesis. Differential effects may also be predicted for the two genders of the same species (as we proposed for human STIs), and this could also be correlated with the probability of STI-induced infertility in the two sexes.
3. The targeted pathomechanisms of infertility could be further investigated using comparative phylogenetic analyses of the pathogen species. The adaptive hypothesis postulates that specific pathomechanisms evolved as the pathogens adapted to sexual transmission: these traits should therefore appear coincident with the switch to sexual transmission in the phylogenetic trees including an STI pathogen and its closely related non-STI sister taxa. E.g., the sexually transmitted lineages (serovars) of *C. trachomatis* could be compared with the non-STI serovars associated with trachoma, or with *C. pneumoniae* [65]. Currently available data only allowed for comparisons with distantly related species (Box 2), but future research is likely to open possibilities for more informative comparisons.
4. Finally, more clinical and laboratory research is needed to expand our general knowledge on the impact of human (and nonhuman) STIs on fertility. Even with the most studied STIs, quantitative estimates of infertility are rare. We were only able to draw quantitative estimates for *N. gonorrhoeae* and *C. trachomatis*, and even in these, only for women, and we had to resort to extrapolating the probability of infertility following infection from the probability of PID in these STIs and the general probability of infertility following PID. However, it is unknown whether PID cases of different aetiology differ in the probability of inducing infertility. Quantitative data would also be needed for the impact of other STIs, including the (quantitative) effect of the infections on male fertility.
